# Impact of US government funding freezes on the HIV response: findings from a rapid survey in 32 countries

**DOI:** 10.1093/haschl/qxag020

**Published:** 2026-02-23

**Authors:** Ellen Brazier, Stephany N Duda, Jeremy Ross, Aggrey S Semeere, Thierry Tiendrebeogo, Cleophas Chimbetete, Denis Nash

**Affiliations:** Institute for Implementation Science in Population Health, City University of New York, Graduate School of Public Health and Health Policy, New York, NY 10027, United States; Department of Biomedical Informatics, Vanderbilt University Medical Center, Nashville, TN 37203, United States; TREAT Asia/amfAR—The Foundation for AIDS Research, Bangkok, 10110 Thailand; Infectious Diseases Institute, College of Health Sciences, Makerere University, Kampala, Uganda; National Institute for Health and Medical Research (INSERM) UMR 1219, Research Institute for Sustainable Development (IRD) EMR 271, Bordeaux Population Health Research Centre, University of Bordeaux, Bordeaux, 33076 France; Newlands Clinic, Harare, Zimbabwe; Institute for Implementation Science in Population Health, City University of New York, Graduate School of Public Health and Health Policy, New York, NY 10027, United States; Department of Epidemiology and Biostatistics, CUNY Graduate School of Public Health and Health Policy, New York, NY 10027, United States

**Keywords:** HIV care, clinic operations, service delivery, US government, funding disruptions

## Abstract

**Introduction:**

After the freezing of US foreign assistance in January 2025, the International Epidemiology Databases to Evaluate AIDS (IeDEA) conducted a rapid survey among clinics and programs actively participating in the research consortium to assess the status of HIV-related service delivery in mid-2025.

**Methods:**

In June–July 2025, an online survey was distributed to 103 IeDEA-participating clinics/programs in 41 countries in sub-Saharan Africa, Latin America and the Caribbean, and Asia-Pacific. Survey domains included US funding-related disruptions in service delivery, medication availability, laboratory services, clinic operations, and attendant mitigation strategies. Responses were received from 76/103 (74%) clinics/programs in 32/41 (78%) countries. Descriptive statistics were used to characterize disruptions in HIV-related care and mitigation measures.

**Results:**

Almost half of responding sites reported disruptions in HIV-related services (47%) since January 2025, along with disruptions in medication availability (28%), laboratory services (34%), and non-research clinic operations (47%). Among those reporting disruptions in HIV-related services, only 14% reported that all disruptions were fully resolved at the time of the survey.

**Conclusion:**

Given anticipated reductions in global HIV funding, government- and community-led monitoring of HIV prevention and treatment services and care outcomes are critical for informing national responses and averting reversal of progress in ending the epidemic.

Key Points

- Among diverse HIV clinics and program across 32 countries in sub-Saharan Africa, Latin America, and Asia-Pacific regions, almost half reported disruptions in HIV-related services after the US government's freezing of foreign assistance in late January 2025.
- More than one-quarter of clinics/programs reported disruptions in HIV counseling and testing, and 16%-24% reported disruptions in HIV treatment, pediatric HIV diagnostic and treatment services, prevention of vertical (ie, mother-to-child) transmission, the provision of PrEP for HIV prevention, and TB treatment.
- Of those experiencing disruptions, few reported full resolution of these issues by the time of the survey in mid-2025.

## Introduction

The freezing of US foreign assistance on January 20, 2025, marked a pivotal shift in the funding landscape for global health, including HIV care and treatment. At the time of the funding freeze, the US President's Emergency Plan for AIDS Relief (PEPFAR) was supporting life-sustaining antiretroviral therapy (ART) for an estimated 20.6 million people with HIV and reaching almost 90 million with HIV testing, pre-exposure prophylaxis (PrEP), or comprehensive HIV prevention services, annually.^1^ While a limited waiver, granted in February 2025, permitted PEPFAR to resume certain “life-saving HIV services,” such as prevention of vertical transmission, PrEP for pregnant and breastfeeding women, and HIV testing,^2^ delays in necessary authorizations and funding uncertainties resulted in disruptions in HIV-related programs, including clinic closures, staff layoffs, and interruptions in HIV-related service delivery, ART drug supplies, and research activities.^3-8^ Modeling studies estimate that PEPFAR funding disruptions resulted in more than 120 000 deaths by November 2025, including more than 13 000 child deaths.^9^ Modeling studies suggest that the termination of PEPFAR-supported programs and US-funded tuberculosis (TB) programs could result in 6.6-8.7 million new HIV cases and 4.2-6.3 million AIDS-related deaths by 2029,^10^ and up to an 10.6 million TB cases and 2.2 million TB deaths by 2030.^11^

To assess the evolving situation for HIV prevention and treatment, the International epidemiology Databases to Evaluate AIDS (IeDEA) research consortium undertook a rapid cross-sectional survey in mid-2025 among a diverse global cohort of HIV clinics and programs in sub-Saharan Africa (SSA), Latin America and the Caribbean (LAC), and the Asia-Pacific region. Our study aimed to assess the status of HIV-related care and services across IeDEA, which includes both PEPFAR-supported and non-PEPFAR-supported countries.

## Study data and methods

Informed by other situational analyses undertaken at national, clinic, and community levels in the immediate wake of the US government's freezing of foreign assistance,^3-6,12,13^ our survey explored whether selected HIV-related services, medication supplies, laboratory services, and clinic operations had been “disrupted or suspended since January 2025 because of changes in US government policy or funding.” For each domain, response options included “Yes,” “No,” “Do not know,” and “Not applicable” for services not provided at the clinic. For each disruption reported, we explored whether or not it was “fully resolved” by the time of the survey. Additionally, we explored the introduction of selected mitigation strategies (eg, expansion of multi-month dispensing [MMD], cost-sharing by clients/patients, telemedicine, and new partnerships with other organizations to ensure service provision for specific groups) in response to funding disruptions.

Designated as non-human subjects research by Vanderbilt University Medical Center's IRB (#250836), the survey was launched on June 1, 2025, as an online REDCap questionnaire,^14^ programmed in English, French, and Spanish. Survey links were distributed to 92 HIV clinics across IeDEA and 11 large HIV programs in East and Southern Africa that represented 198 individual clinics (range: 2 to 42 clinics per program). Comprising all sites in SSA, LAC, and Asia-Pacific regions that were actively participating in IeDEA in 2025, these 103 clinics and programs were serving an estimated population of 535 700 with HIV care and treatment services at the beginning of 2025.

The survey closed on July 31, 2025, with responses from 68 individual clinics (74%) and 8 programmatic cohorts (73%) across 32 countries in East, Central, Southern, and West Africa, Latin America, and the Asia-Pacific. Clinics and programs were weighted equally in the calculation of descriptive statistics.

## Results

**Figure 1.**
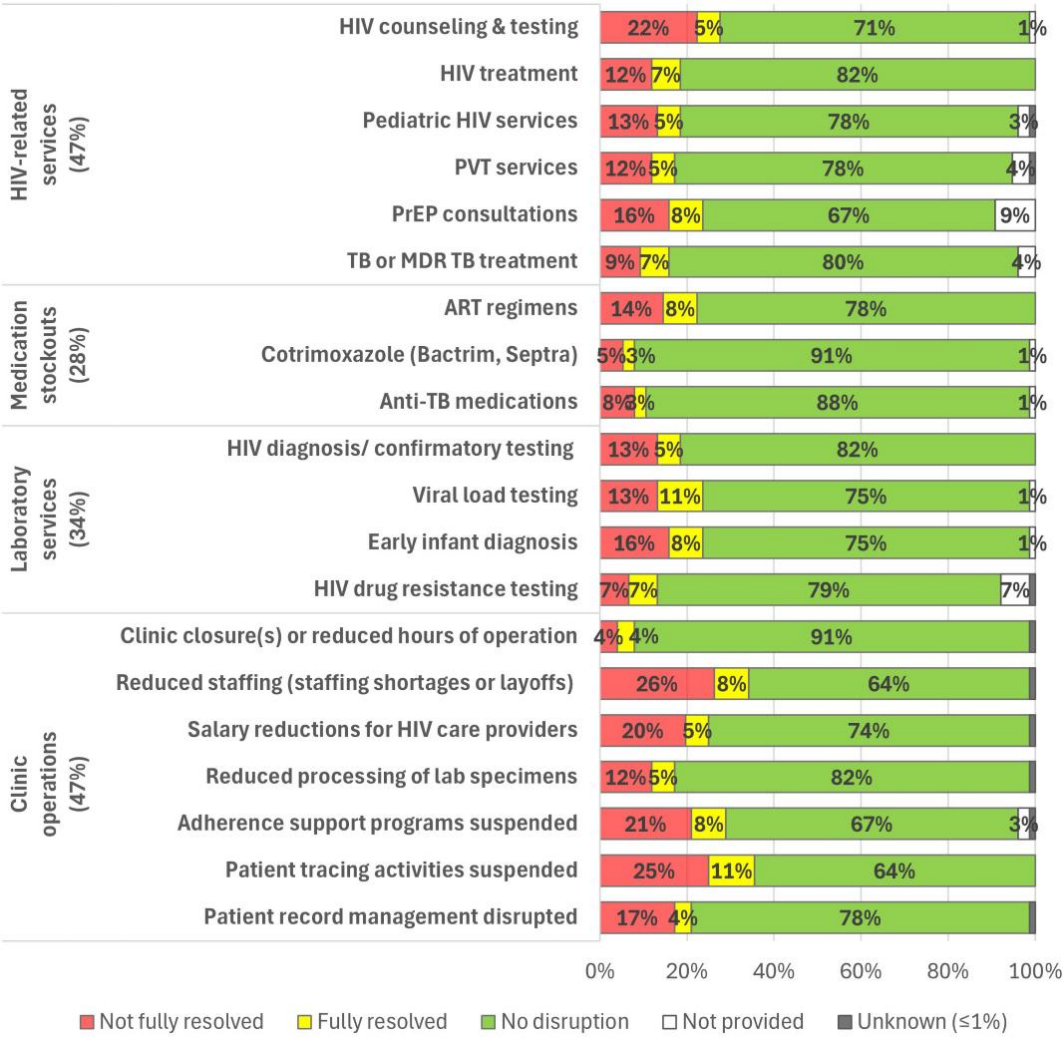
US government funding-related disruptions HIV-related services and clinic operations reported by 76 IeDEA clinics and programs* and their resolution status as of June-July, 2025. Abbreviations: ART, antiretroviral therapy; MDR-TB, multi-drug-resistant tuberculosis; PrEP, pre-exposure prophylaxis; TB, tuberculosis. *Clinics and programs were weighted equally in the calculation of descriptive statistics.

Most responding sites were located within hospitals (58%), urban settings (86%), and countries receiving PEPFAR support at the beginning of 2025 (88%). Almost half (47%) reported clinic-level disruptions in HIV-related service delivery since January 2025, with 27% reporting disruptions in HIV counseling and testing (Figure 1), and 16%-24% reported disruptions in HIV treatment, pediatric HIV services, prevention of vertical (ie, mother-to-child) transmission (PVT), provision of PrEP, TB treatment or other services. Among those reporting any service delivery disruptions, 14% reported that all disruptions were fully resolved by the time of survey completion.

**Figure 2.**
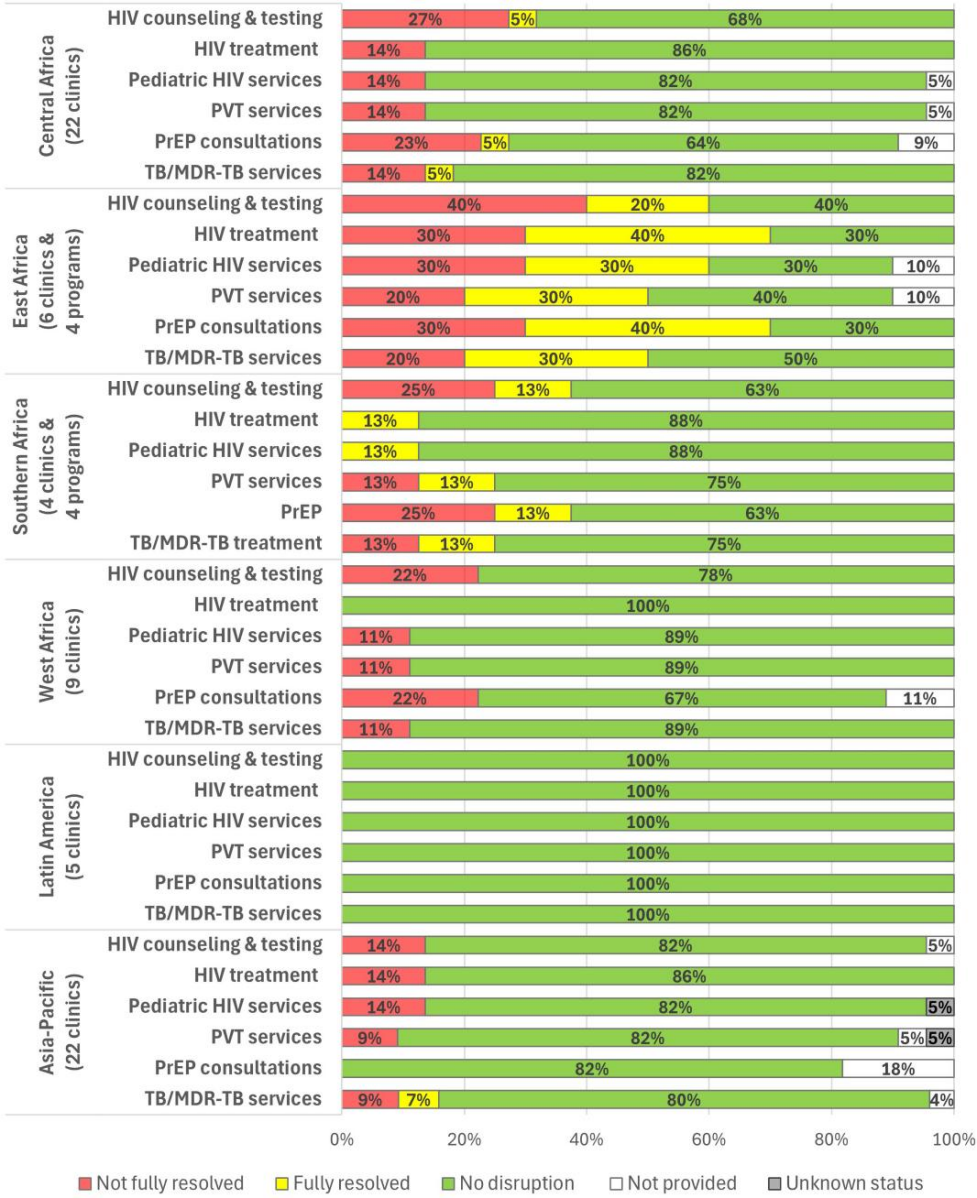
Disruptions in HIV-related services reported by 76 IeDEA clinics and programs* and their resolution status as of June–July, 2025, by IeDEA region. Abbreviations: ART, antiretroviral therapy; MDR-TB, multi-drug-resistant tuberculosis; PrEP, pre-exposure prophylaxis; PVT, prevention of vertical transmission; TB, tuberculosis. *Clinics and programs were weighted equally in the calculation of descriptive statistics.

Disruptions in HIV-related service delivery and their resolution by the survey timepoint varied across IeDEA regions, with no sites in Latin America reporting any disruption in HIV-related services, compared with a majority of clinics/programs in East Africa (Figure 2). In most regions, HIV counseling and testing and PrEP were the most frequently reported disruptions, and in Central Africa, West Africa, and Asia-Pacific, none of those reporting disruptions in HIV treatment, pediatric HIV services, or PVT indicated that they were fully resolved by the survey timepoint.

Approximately one-quarter (28%) of clinics/programs reported disruptions in the availability of essential medications since January 2025, ranging from 0% of responding sites in Latin America to 50% in East Africa. Overall, 22% reported stockouts or shortages of ART medications, and 11% and 8%, respectively, reported stockouts/shortages of anti-TB medications and broad-spectrum antibiotics. Of those reporting medication shortages/stockouts, only 19% reported they were fully resolved at the time of the survey.

One-third (34%) reported disruptions in laboratory services since January 2025 (range: 0% of respondents in Latin America to 80% in East Africa). Disruptions included HIV diagnosis/confirmatory testing (18%), early infant diagnosis (24%), viral load testing (24%), and HIV drug resistance testing (14%), with only 31% reporting that all disruptions in laboratory services were fully resolved by the time of the survey.

Almost half (47%) reported disruptions in other clinic operations related to HIV care, ranging from 44% of responding sites in West Africa to 70% in East Africa. Such disruptions included staffing shortages or layoffs (33%), salary reductions for HIV providers (24%), the suspension of patient tracing activities (33%) and adherence support programs (29%), and disruptions in patient record management (21%) and laboratory processing of specimens (17%). Additionally, 42% reported disruption or suspension of research.

Disruptions in HIV-related service delivery (52%), medication availability (31%), laboratory services (37%), and non-research clinic operations (51%) were more prevalent among clinics and programs in PEPFAR-supported countries, compared with non-PEPFAR countries (11%, 0%, 11%, and 22%, respectively).

The most common mitigation measures introduced in response to the US funding freeze were the expansion of MMD (21% of all clinics/programs, ranging from 0% in Latin America to 60% in East Africa), followed by the introduction of cost-sharing by clients/patients (reported by 14% of responding sites in Central Africa and Asia-Pacific regions). Increased use of telemedicine was reported by 5% of clinics/programs, and 11% reported new partnership(s) with other organizations to ensure service delivery.

## Discussion

Consistent with other reports on the immediate impacts of the US government's freezing of foreign assistance,^4,7^ our rapid survey of HIV clinics and programs across 32 countries revealed disruptions in HIV-related services and other clinic operations after January 2025, with few disruptions fully resolved by the survey timepoint in mid-2025. The extent of disruptions and their full resolution varied substantially across IeDEA's regions and by service type; disruptions in clinic services and non-research operations were more prevalent among clinics/programs in East and Southern Africa, and, in these regions, some reported they were fully resolved at the survey timepoint. In contrast, few clinics in Central and West Africa and Asia-Pacific regions reporting disruptions indicated that they were fully resolved by mid-2025. While the concentration of service delivery disruptions in PEPFAR-supported countries is expected, service delivery disruptions reported by one clinic in a non-PEPFAR country could reflect changes in the activities of international NGOs supporting the HIV response, services that had been supported through clinical research, or government allocations related to anticipated changes in disbursements from The Global Fund.

We observed larger and ongoing disruptions in service areas such as PrEP, HIV counseling and testing, laboratory services, and support functions (eg, adherence support, patient tracing, record-keeping, and clinic staffing), compared with HIV treatment and PVT services, which may reflect clearer guidance on the continuation of the latter services under the February 2025 limited waiver. Nonetheless, among our sample, disruptions in these services appeared more pervasive and persistent in high HIV burden settings than acknowledged by the US government, which asserted in May 2025 that PEPFAR remained “85% operative” in terms of delivering HIV care and treatment services.^15^ While core HIV treatment services supported by PEPFAR (eg, commodities, supplies, and staffing) are crucial in reaching epidemic control, disruptions in prevention and support services for key and high-risk populations may also jeopardize progress in many settings.^16^

Our findings should be interpreted in the context of several limitations. Clinics and programs participating a global research consortium, such as IeDEA, may not be nationally or regionally representative, and may not reflect the status of small, rural clinics not involved in research. Additional sources of selection bias include the possibility that clinics affected by the funding freeze were more likely to participate than less-affected clinics, potentially inflating our estimates. However, it is also possible that clinics with severe disruptions (eg, clinic closures, staffing shortages, etc.) were less likely to participate, potentially underestimating disruptions.

To minimize response burden and potential recall biases, our cross-sectional survey did not explore the exact timing of disruptions and their resolution, participating clinics' sources of funding and support, changes in service quality resulting from disrupted clinic operations, or patient-level outcomes. The impacts of the US funding freeze may have varied over time and across PEPFAR implementing partners in important ways our survey could not capture. Additionally, as PEPFAR funding does not support all HIV clinics in PEPFAR-supported countries, our estimates may not accurately reflect the impact of US funding freezes in these settings.

While our survey was completed by seasoned partners who routinely complete IeDEA's periodic site assessments,^17^ several potential response biases are important to acknowledge. It is possible that those completing the survey lacked complete information on the status of service delivery and clinic operations or were concerned that reporting on disruptions could jeopardize clinic or program funding. Additionally, our survey may be subject to negative response biases if respondents were personally affected by salary reductions, increased workload, or other adverse impacts of the funding freeze, or if they expected that IeDEA might be positioned to mitigate the disruptions they reported.

Finally, where the survey was completed on behalf of a program comprised of multiple clinics, responses may obscure important variation across clinics, and our equal weighting of clinic and program responses may under- or overestimate the impacts of US funding disruptions on patient-level outcomes, with disruptions among programs serving large populations being most consequential.

These limitations notwithstanding, our study provides a snapshot of HIV-related service delivery across diverse settings in mid-2025—a period with limited empirical evidence on the status of the global HIV response. Our findings highlight critical aspects of the HIV response that will be important to monitor as government-to-government agreements take shape under the new America First Global Health Strategy—a strategy that prioritizes funding for health commodities and frontline workers and promises decreased funding for other areas.^18,19^ Anticipated reductions in global funding for HIV may ultimately catalyze stronger and more resilient national responses, including strengthened country ownership, the integration of HIV services into primary health care, improved efficiency, increased domestic resource mobilization, and reduced donor dependency.^20-23^ In the interim, government- and community-led monitoring of the availability, accessibility, and quality of HIV prevention and care and tracking patient outcomes along the care continuum will be essential for identifying and responding to emerging gaps in the HIV response in countries where international aid has been instrumental in recent gains toward ending the epidemic.

## Supplementary Material

qxag020_Supplementary_Data
