# Supplementary material for: Impact of US government funding freezes on the HIV response: findings from a rapid survey in 32 countries
Source: Health Aff Sch. 2026 Feb 23;4(2):qxag020. doi: 10.1093/haschl/qxag020 (PMC12925645; doi:10.1093/haschl/qxag020)
Supplement: qxag020_Supplementary_Data [file qxag020_supplementary_data.zip › SupplementaryMaterials.docx]

**Funding Acknowledgements**

The International Epidemiology Databases to Evaluate AIDS (IeDEA) is supported by the U.S. National Institutes of Health’s (NIH) National Institute of Allergy and Infectious Diseases, the *Eunice Kennedy Shriver* National Institute of Child Health and Human Development, the National Cancer Institute, the National Institute of Mental Health, the National Institute on Drug Abuse, the National Heart, Lung, and Blood Institute, the National Institute on Alcohol Abuse and Alcoholism, the National Institute of Diabetes and Digestive and Kidney Diseases, and the Fogarty International Center: ***Asia-Pacific***, U01AI069907; ***CCASAnet***, U01AI069923; ***Central Africa***, U01AI096299; ***East Africa***, U01AI069911; ***NA-ACCORD***, U01AI069918; ***Southern Africa***, U01AI069924; ***West Africa***, U01AI069919. Informatics resources are supported by the Harmonist project, R24AI124872. This publication is the result of funding in whole or in part by the NIH. It is subject to the NIH Public Access Policy. Through acceptance of this federal funding, NIH has been given a right to make this manuscript publicly available in PubMed Central upon the Official Date of Publication, as defined by NIH. The content of this publication is solely the responsibility of the authors and does not necessarily represent the official views of any of the governments or institutions mentioned above.

**Asia-Pacific**

**The TREAT Asia HIV Observational Database**

V Khol, V Ouk, C Pov, V bun, National Center for HIV/AIDS, Dermatology & STDs, Phnom Penh, Cambodia; MP Lee, PCK Li, TS Kwong, KY Yeung, Queen Elizabeth Hospital, Hong Kong SAR, China; N Kumarasamy, S Poongulali, B Faith, VHS-Infectious Diseases Medical Centre, Chennai Antiviral Research and Treatment Clinical Research Site (CART CRS), Voluntary Health Services, Chennai, India; S Pujari, K Joshi, S Gaikwad, A Chitalikar, Institute of Infectious Diseases, Pune, India; H Prasad, V Mave, I Marbaniang, S Nimkar, BJ Government Medical College and Sassoon General Hospital, Pune, India; IKA Somia, TP Merati, NM Dewi Dian Sukmawati, F Yuliana, Faculty of Medicine Udayana University - Ngoerah Hospital, Bali, Indonesia; E Yunihastuti, B Wicaksana, A Widhani, S Maria, Faculty of Medicine Universitas Indonesia - Dr. Cipto Mangunkusumo General Hospital, Jakarta, Indonesia; J Tanuma, H Gatanaga, H Uemura, Y Koizumi, National Center for Global Health and Medicine, Tokyo, Japan; JY Choi, JH Kim, JE Park, Division of Infectious Diseases, Department of Internal Medicine, Yonsei University College of Medicine, Seoul, South Korea; YM Gani, TK Heng, NA Misnan, SK Chidhambaram, Hospital Sungai Buloh, Sungai Buloh, Malaysia; I Azwa, A Kamarulzaman, SF Syed Omar, S Ponnampalavanar, University Malaya Medical Centre, Kuala Lumpur, Malaysia; RA Ditangco, MK Pasayan, JB Sornillo, Research Institute for Tropical Medicine, Muntinlupa City, Philippines; HP Chen, YJ Chan, PF Wu, Taipei Veterans General Hospital, Taipei, Taiwan; CY Choy, PH Lee, PA Kumar, Z Ferdous, Tan Tock Seng Hospital, Singapore; A Avihingsanon, N Hiranburana, C Wongvoranet, C Ruengpanyathip, HIV-NAT/Thai Red Cross AIDS and Infectious Diseases Research Centre, Bangkok, Thailand; S Kiertiburanakul, A Phuphuakrat, L Chumla, N Sanmeema, Faculty of Medicine Ramathibodi Hospital, Mahidol University, Bangkok, Thailand; R Chaiwarith, T Sirisanthana, J Praparattanapan, K Nuket, Faculty of Medicine and Research Institute for Health Sciences, Chiang Mai University, Chiang Mai, Thailand; S Khuwuwan, P Kambua, S Pongrapass, J Limlertchareonwanit, Chiangrai Prachanukroh Hospital, Chiang Rai, Thailand; TN Pham, KV Nguyen, DTH Nguyen, DT Nguyen, National Hospital for Tropical Diseases, Hanoi, Vietnam; CD Do, AV Ngo, LT Nguyen, Bach Mai Hospital, Hanoi, Vietnam; AH Sohn, JL Ross, B Petersen, TREAT Asia, amfAR - The Foundation for AIDS Research, Bangkok, Thailand; K Petoumenos, A Jiamsakul, D Rupasinghe, The Kirby Institute, UNSW Sydney, NSW, Australia.

**IeDEA Caribbean, Central, and South America (CCASAnet)**

**Fundación Huésped, Argentina:** Pedro Cahn, Carina Cesar, Valeria Fink, Florencia Cahn, Ines Aristegui, Maria Ines Figueroa, Nicolas Doudtchitzky, Javier Mariani, Mariela Ceschel, Gissella Mernies, Agustin Nava.

**Instituto Nacional de Infectologia-Fiocruz, Brazil:** Beatriz Grinsztejn, Valdilea G. Veloso, Paula M. Luz, Sandra Wagner Cardoso, Ruth Friedman, Ronaldo I. Moreira,Monica Derrico Pedrosa, Hugo Perazzo, Rodrigo Moreira, Maria Pia Diniz Ribeiro, Mario Sergio Pereira, Emilia Moreira Jalil, Thiago Silva Torres, Carolina Coutinho, Mayara Secco Torres Silva,  Flaviana Pavan Victoriano

**Universidade Federal de Minas Gerais, Brazil:** Jorge Pinto, Flavia Ferreira, Marcelle Maia, Victoria Bocardi, Julia Caporali, Flavia Fonseca.

**Universidade Federal de São Paulo, Brazil:** Regina Célia de Menezes Succi, Daisy Maria Machado, Aida de Fátima Barbosa Gouvêa, Fabiana Bononi do Carmo.

**Fundación Arriarán & Universidad de Chile, Chile:** Claudia P. Cortes, Maria Fernanda Rodriguez, Gabriel Castillo-Rozas, Fabio Paredes, Georgina Estadella.

**Les Centres GHESKIO, Haiti:** Jean William Pape, Vanessa Rouzier, Adias Marcelin, Jodany Bernadin, Stanley Cadet.

**Hospital Escuela Universitario, Honduras**: Marco Tulio Luque, Diana Varela, Magda Chavez, Ada Mailhot.

**Instituto Hondureño de Seguridad Social, Honduras:** Marco Tulio Luque.

**Instituto Nacional de Ciencias Médicas y Nutrición Salvador Zubirán, Mexico**: Brenda Crabtree Ramirez, Juan Sierra Madero, Yanink Caro Vega, Álvaro López Iñiguez, Ana Fernanda Ramos Menchelli, Paola Alarcón Murra, Geovanna Coello, Guadalupe Muñuzuri Nájera, Rodrigo Ville Benavides, Jessica Mejía, Távata Bejarano, Nancy Sierra.

**Instituto de Medicina Tropical Alexander von Humboldt, Universidad Peruana Cayetano Heredia, Peru**: Eduardo Gotuzzo, Fernando Mejia, Gabriela Carriquiry, Claudia Nuñez Mochizaki.

**Vanderbilt University Medical Center, USA:** Jessica L Castilho, Stephany N Duda, Bryan E Shepherd, Timothy R Sterling, Anna K Person, Peter F Rebeiro, William C Wester, Karu Jayathilake, John Koethe, Rachael A Pellegrino, Fernanda Maruri, Hilary Vansell Riley, Amanda Garcia De Matos Amaral, Marina Cruvinel Figueiredo, Paridhi Ranadive, Megan Turner, Gustavo Amorim, Felipe Ridolfi, Vickie Myers, Heather Burgess, Liping Du, Jesse Carlson, Danni Shi, Zhouhui Liang, Kaixing Liu, Chao Yan

**Central Africa IeDEA**

**Site investigators and cohorts:** Nimbona Pélagie, Annabelle Niyongabo, Association Nationale de Soutien aux Séropositifs et Malades du Sida (ANSS),Burundi; Jérémie Biziragusenyuka, Jeanine Munezero, Valentin Nitereka, Jean Marie Ntibigarura, Christella Twizere, Centre National de Référence en Matière de VIH/SIDA, Burundi; Hélène Bukuru, Thierry Nahimana, Martin Manirakiza, Centre de Prise en Charge Ambulatoire et Multidisciplinaire des PVVIH/SIDA du Centre Hospitalo-Universitaire de Kamenge (CPAMP-CHUK), Burundi; Patrice Barasukana, Hélène Bukuru, Martin Manirakiza, Burundi National University, Burundi; Ella Ange Kazigamwa, Marie-Francine Uwamahoro, Centre de Prise en Charge Ambulatoire et Multidisciplinaire des PVVIH/SIDA de l’Hôpital Prince Régent Charles (CPAMP-HPRC), Burundi; Jeanne Marie Francine Karemera, Populations Services International, Burundi; Jérémie Biziragusenyuka, U.S Embassy, Burundi; Ernestine Kesah, Christabelle Ewane, Denis Nsame, Vera Veyieeneneng, Bamenda Regional Hospital, Cameroon; Rogers Ajeh, Dan Ebai Ashu, Eta Atangba, Jordanne Ching, Christelle Tayomnou Deussom, Peter Vanes Ebasone, Emmanuel Gwan, Ernestine Kendowo, Clarisse Lengouh, Sandra Mimou Mbunguet, Judith Nasah, Eric Ngassam, Antoinette Nsam-Akum, Evodia Tibah, Dorcas Cheko, Clenise Ngwa, Anyangwa Sidonie, Clinical Research Education and Consultancy (CRENC), Cameroon; Anastase Dzudie, CRENC and Douala General Hospital, Cameroon; Djenabou Amadou, Ekoua Daniel, Eric Pefura Yone, Jamot Hospital, Cameroon; Annereke Nyenti, Phyllis Fon, Mary Adiah, Priscilia Enow, Limbe Regional Hospital, Cameroon; Catherine Akele, Akili Clever, Faustin Kitetele, Patricia Lelo, Kalembelembe Pediatric Hospital, Democratic Republic of Congo; Guy Nkoba, Martine Tabala, Cherubin Ekembe, Pélagie Babakazo, Kinshasa School of Public Health, Democratic Republic of Congo; Jean Paul Nzungani, Viviane Diankenda, Dany Lukeba, Sangos plus/Bomoi, Democratic Republic of Congo; Mattieu Musiku, Therese Mpongo, Job Nsoki, Hopital de Kabinda, Democratic Republic of Congo; Merlin Diafouka, Dominique Mahambou Nsonde, CTA Brazzaville, Republic of Congo; Ursula Koukha, Adolphe Mafoua, Massamba Ndala Christ, CTA Pointe-Noire, Republic of Congo; Jules Onesphore Igirimbabazi, Nicole Ayinkamiye, Bethsaida Health Center, Rwanda; Providance Uwineza, Emmanuel Ndamijimana, Busanza Health Center, Rwanda; Jean Baptiste Habumuremyi, Jean Marie Vianney Barinda, Marie Louise Nyiraneza, Gahanga Health Center, Rwanda; Marie Louise Nyiransabimana, Liliane Tuyisenge, Gikondo Health Center, Rwanda; Epiphanie Mukashyaka, Catherine Kankindi, Christian Shyaka, Kabuga Health Center, Rwanda; Bonheur Uwakijijwe, Marie Grace Ingabire, Kicukiro Health Center, Rwanda; Berthilde Uwamariya, Jules Ndumuhire, Masaka Health Center, Rwanda; Isaac Rushimisha, Gerard Bunani, Fred Muyango, Nyagasambu Health Center, Rwanda; Wilbrold Habiyaremye, Yvette Ndoli, Oliver Uwamahoro, Nyarugunga Health Center, Rwanda; Eugenie Mukashyaka, Rosine Feza, Shyorongi Health Center, Rwanda; Chantal Benekigeri, Jacqueline Musaninyange, WE-ACTx for Hope Clinic, Rwanda; Josephine Gasana, Jocelyne Ingabire, Faustin Kanyabwisha, Gallican Kubwimana, Fabiola Mabano, Eric Manzi, Jean Paul Mivumbi, Benjamin Muhoza, Verene Mukankurunziza, Gad Murenzi, Francoise Musabyimana, Allelluia Giovanni Ndabakuranye, Mary Gertrude Rutwaza, Diane Ryumugabe, Patrick Tuyisenge, Vanessa Umuhoza, Francine Umwiza, Research for Development (RD Rwanda), Rwanda; Jules Kabahizi, Janviere Mutamuliza, Boniface Nsengiyumva, Ephrem Rurangwa, Rwanda Military Hospital, Rwanda; Eric Remera, Gallican Nshogoza Rwibasira, Rwanda Biomedical Center, Rwanda.

**Coordinating and Data Centers:** Kathryn Anastos, Abena Bosompem, Jean Claude Dusingize, Viraj Patel, Jonathan Ross, Marcel Yotebieng, Natalie Zotova, Albert Einstein College of Medicine, USA; Ryan Barthel, Ellen Brazier, Denis Nash, Chloe Teasdale, Institute for Implementation Science in Population Health, Graduate School of Public Health and Health Policy, City University of New York (CUNY), USA; Batya Elul, Columbia University, USA; Xiatao Cai, Don Hoover, Qiuhu Shi, Data Solutions, USA; Mark Kuniholm, University at Albany, State University of New York, USA; Andrew Edmonds, Angela Parcesepe, Jess Edwards, University of North Carolina at Chapel Hill, USA; Olivia Keiser, University of Geneva; Stephany Duda; Vanderbilt University School of Medicine, USA; April Kimmel, Virginia Commonwealth University School of Medicine, USA; Adebola Adedimeji, Kathryn Lancaster, Wake Forest Health, USA.

**East Africa IeDEA**

**Site investigators and cohorts:** Lameck Diero, Ann Mwangi, Anthony Ngeresa, MOI University, Academic Model Providing Access To Healthcare (AMPATH), Eldoret, Kenya; Elizabeth Bukusi, Francesca Odhiambo, Raphael Onyango, Wycliffe Opande, Kenya Medical Research Institute, Centre for Microbiology-Research Care and Training Program (KEMRI-CMR-RCTP), Kisumu, Kenya;, Kisumu, Kenya; Charles Kasozi, Mathew Ssemakadde, Masaka Regional Referral Hospital, Masaka, Uganda; Winnie Muyindike, Helen Byakwaga, Bronia Mwiine Kwarisiima, David Muhumuza, Mbarara University of Science and Technology (MUST), Mbarara, Uganda; Barbara Castelnuovo, John Michael Matovu, Infectious Diseases Institute (IDI), Makerere University, Kampala, Uganda; Fred Nalugoda, Anthony Ndyanabo, Rakai Health Sciences Program (RHSP), Kalisizo, Uganda; Paul Kazyoba, Mary Mayige, Tanzanian Institute of Medical Research (NIMR), Dar es Salaam, Tanzania; Rita Elias Lyamuya, Godloveness Rubega, Morogoro Regional Hospital, Morogoro, Tanzania; Happiness Edward Rutakulemberwa, Jerome Lwali, Tumbi Regional Hospital, Pwani, Tanzania; Denna Michael, Richard Machemba, NIMR, Kisesa HDSS, Mwanza, Tanzania; Jimmy Carlucci, Megan McHenry, Harold Kooreman, Jacob Leighty, Michael Menser, Indiana University, Indianapolis, USA; Batya Elul, Columbia University, New York City, NY, USA; Neelima Navuluri, Duke University, Durham, NC, USA; Rachel Vreeman, Mt. Sinai, New York, USA; Jeffrey Martin, Megan Wenger, Craig Cohen, Jayne Kulzer, University of California, San Francisco, CA, USA; Rena Patel, University of Alabama at Birmingham, Birmingham, USA

**East Africa IeDEA Regional Data Center:** Leslie Enane, John Humphrey, Beverly Musick, and Kara Wools-Kaloustian, Indiana University, Indianapolis, USA; Ann Mwangi and Edwin Sang, Moi University, Eldoret, Kenya: Aggrey Semeere, Infectious Diseases Institute (IDI), Makerere University, Kampala, Uganda; Constantin Yiannoutsos, The City University of New York (CUNY), New York, USA.

**IeDEA Southern Africa**

**Site investigators and cohorts:** Gary Maartens, Aid for AIDS, South Africa; Carolyn Bolton/Guy Kayeya Muula, Centre for Infectious Disease Research in Zambia (CIDRZ), Zambia; Robin Wood, Gugulethu (Desmond Tutu HIV Centre), South Africa; Nosisa Sipambo, Harriet Shezi Children’s Clinic, South Africa; Frank Tanser, Hlabisa (Africa Health Research Institute), South Africa; Andrew Boulle/Jonathan Euvrard, Khayelitsha ART Programme, South Africa; Geoffrey Fatti, Kheth’Impilo AIDS Free Living, South Africa; Ethel Rambiki, Lighthouse Trust, Malawi; Mazvita Muchengeti, National Cancer Registry (National Health Laboratory Service), South Africa; Cleophas Chimbetete, Newlands Clinic (Ruedi Luethy Foundation Zimbabwe), Zimbabwe; Karl Technau, Rahima Moosa Mother and Child Hospital, South Africa; Brian Eley, Red Cross War Memorial Children’s Hospital, South Africa; Irene Ayakaka, SolidarMed Lesotho; Idiovino Rafael, SolidarMed Mozambique; Cordelia Kunzekwenyika, SolidarMed Zimbabwe; Matthew P Fox, Themba Lethu Clinic, South Africa; Hans Prozesky, Tygerberg Hospital, South Africa; Andrew Boulle, Western Cape HIV and TB Cohort, South Africa.

**Data centers:** Nanina Anderegg, John Andoh, Marie Ballif, Nicolas Banholzer, Cam Ha Dao Ostinelli, Matthias Egger, Lukas Fenner, Nathalie Fernandez, France Genin, Andreas Haas, Nicola Low, Ardele Mandiriri Ndanga, Eliane Rohner, Carlotta Riebensam, Yann Ruffieux, Remo Schmutz, Katayoun Taghavi, Per von Groote, Gilles Wandeler, Institute of Social and Preventive Medicine, University of Bern, Switzerland; Kim Anderson, Andrew Boulle, Chido Chinogurei, Mary-Ann Davies, Shani De Beer, Leigh Johnson, Reshma Kassanjee, Nicola Maxwell, Haroon Moolla, Patience Nyakato, Gem Patten, Mpho Tlali, Renee de Waal, Wendy Wiemers, Confidence Mothiba, Emma Kalk, The Centre for Integrated Data and Epidemiological Research, School of Public Health, University of Cape Town, South Africa.

**IeDEA West Africa**

**Site investigators and cohorts: Adult cohorts**: Marcel Djimon Zannou, CNHU, Cotonou, Benin; Armel Poda, CHU Souro Sanou, Bobo Dioulasso, Burkina Faso; Oliver Ezechi, National Institute of Medical Research (NIMR), Lagos, Nigeria. Eugene Messou, ACONDA CePReF, Abidjan, Cote d’Ivoire; Henri Chenal, CIRBA, Abidjan, Cote d’Ivoire; Kla Albert Minga, CMSDS, Abidjan, Cote d’Ivoire; Aristophane Tanon, CHU Treichville, Cote d’Ivoire; Moussa Seydi, CHNU de Fann, Dakar, Senegal; Ephrem Mensah, Clinique EVT, Lomé, Togo. Bem Tar, Federal Medical Center, Makurdi, Nigeria; James Samuel, Jos University Teaching Hospital, Nigeria; Muyiwa Ojo-osagie, University College Hospital, Ibadan, Nigeria; Azeez Ayotunde Federal Medical Center, Abeokuta, Nigeria; Sheriff, Bolanle Yemisi Alabi, State Specialist Hospital, Akure, Nigeria. **Pediatric cohorts**: Caroline Yonaba, CHU Yalgado Ouadraogo; Lehila Bagnan, CNHU, Cotonou, Benin; Jocelyn Dame, Joycelyn Assimeng Dame, Korle Bu Hospital, Accra, Ghana; Sylvie Marie N’Gbeche, ACONDA CePReF, Abidjan, Ivory Coast; Kouadio Kouakou, CIRBA, Abidjan, Cote d’Ivoire; Madeleine Amorissani Folquet, CHU de Cocody, Abidjan, Cote d’Ivoire; François Tanoh Eboua, CHU de Yopougon, Abidjan, Cote d’Ivoire; Mariam SYLLA, Hopital Gabriel Toure, Bamako, Mali; Oliver Ezchechi,, Agatha David, Rosemary Audu, NIMR, Lagos, Nigeria; Elom Takassi, CHU Sylvanus Olympio, Lomé, Togo. Ahula David, Victor Ojeh Bazim, Federal Medical Center, Makurdi, Nigeria; Jos University Teaching Hospital, Nigeria; Olarenwaju Ogunyide, University College Hospital, Ibadan, Nigeria; Mariam Moji Ogundeyi, Federal Medical Center, Abeokuta, Nigeria; Olaseinde Bello, State Specialist Hospital, Akure, Nigeria.

**Regional coordination:** Antoine Jaquet (PI), Didier Koumavi Ekouevi (PI), François Dabis, Charlotte Bernard, Sophie Labarriere, Karen Malateste, Olivier Marcy, Marie Kerbie Plaisy, Elodie Rabourdin, Thierry Tiendrebeogo. ADERA, University of Bordeaux, National Institute for Health and Medical Research (Inserm) UMR1219, Research Institute for Sustainable Development (IRD) EMR 271, Bordeaux Population Health Centre, Bordeaux, France. Désiré Dahourou, Sophie Desmonde, Julie Jesson, Valeriane Leroy, Emile Sodenyessi. CERPOP, Inserm UMR1295, Toulouse, France. Raoul Moh, Jean-Claude Azani, Kadidja Diarra, Jean Jacques Koffi, Maika Bengali, Abdoulaye Cissé, Guy Gnepa, Eric Komena, Apollinaire Horo, Séverin Lenaud, Simon Boni, Eulalie Kangah, Corinne Moh, Jeanne Eliam, PAC-CI program, CHU Treichville, Abidjan, Côte d’Ivoire.

**Partner institutions:** Case Western Reserve University: Igho Ofotokun (PI). Washington University: Noëlle Benzekri, Geoffrey Gottlieb. APIN Public Health Initiatives, Abuja, Nigeria: Prosper Okonkwo, Oluwaseun Olaifa, Uche Okezie.
